# The contribution of BTK signaling in myeloid cells to neuroinflammation

**DOI:** 10.3389/fimmu.2025.1595069

**Published:** 2025-06-18

**Authors:** Claudia Bassani, Marta Molinari, Valentina Romeo, Vittorio Martinelli, Ursula Boschert, Gianvito Martino, Luca Muzio, Cinthia Farina

**Affiliations:** ^1^ Institute of Experimental Neurology and Division of Neuroscience, Istituto di Ricovero e Cura a Carattere Scientifico (IRCCS) San Raffaele Scientific Institute, Milan, Italy; ^2^ The Healthcare Business of Merck KGaA, Darmstadt, Germany

**Keywords:** BTK, evobrutinib, experimental autoimmune encephalomyelitis, monocyte, multiple sclerosis, myeloid cell

## Abstract

**Introduction:**

Bruton’s tyrosine kinase (BTK) is a member of the TEC family of non-receptor tyrosine kinases expressed in cells of hematopoietic origin, including B lymphocytes and myeloid cells. Selective BTK inhibitors (BTKi) have shown efficacy in clinical trials in multiple sclerosis (MS). Here we investigated the role of BTK in human and mouse myeloid cells in *in vitro* and *in vivo* studies.

**Methods:**

We evaluated i) the impact of the BTK inhibitor (BTKi) evobrutinib on monocyte markers for activation, costimulation, adhesion and phagocytosis in peripheral blood mononuclear cell (PBMC) cultures from healthy and MS subjects; ii) the therapeutic effects and the action of evobrutinib on myeloid cell phenotype in the experimental autoimmune encephalomyelitis (EAE) model of MS; iii) the contribution of BTK in short-lived vs. long-lived myeloid cells to EAE expression via experiments with double transgenic mice allowing inducible inactivation of BTK in CX3CR1 expressing cells.

**Results:**

We report that BTKi supported monocyte expression of VLA4/CD49d, an integrin directing immune cell migration towards the central nervous system, and CD163, a well-known scavenger receptor involved in removal of myelin debris, in samples from healthy subjects. This effect was maintained under distinct inflammatory settings and replicated with PBMC of MS subjects. Therapeutic intervention with evobrutinib ameliorated EAE severity and was associated with a significant modest decrease in the frequency of CNS-infiltrating proinflammatory macrophages. However, conditional BTK deletion in short-lived or long-lived CX3CR1-positive cells did not reduce EAE severity.

**Discussion:**

This functional evidence questions the real contribution of BTK expressing myeloid cells to experimental MS.

## Introduction

Tec kinases represent a subfamily of non-receptor protein tyrosine kinases (PTKs) important for intracellular signaling (1). This family consists of five members, among which Bruton’s tyrosine kinase (BTK) is expressed in cells of hematopoietic origin and regulates innate and adaptive immune responses (2, 3). Loss-of-function mutations in this gene may lead to X-linked agammaglobulinemia (XLA), a genetic disorder characterized by a block in B-cell development and, thus, no or little immunoglobulin production (3). Indeed, antigen binding to the B-cell receptor complex triggers recruitment to the plasma membrane and phosphorylation of BTK, which activates PLCγ2 signaling and, thereby, regulates B-cell survival, proliferation, differentiation, and antibody secretion (4). As B cells strongly depend on BTK activity for function and survival, this protein has been used as a target for the development of small molecule inhibitors in B-cell malignancies and autoimmune disorders such as multiple sclerosis (MS), a chronic inflammatory disorder of the central nervous system (CNS) (5, 6). Currently, several Phase II and III trials with distinct BTK inhibitors are ongoing for MS (6), and initial results are available (7, 8). In addition to its crucial role in B-cell signaling, BTK may regulate innate immune reactions downstream of FcgR and Toll-like receptor signaling in myeloid cells (2, 6). Here, we investigated the role of BTK in human and mouse myeloid cells in the context of multiple sclerosis by *in vitro* and *in vivo* studies.

## Materials and methods

### Human subjects

Investigations were conducted according to the principles expressed in the Declaration of Helsinki and after approval of the study by the Ethics Committee of Ospedale San Raffaele. MS subjects were diagnosed according to the McDonald criteria (9) and were not under any disease-modifying therapy. After signed informed consent was provided, peripheral blood was drawn from three neurological patients (male, 36 years old, 9-month disease duration; female, 52 years old, 2-month disease duration; and female, 39 years old, 12-month disease duration) and three healthy controls (male, 40 years old; female, 26 years old; and female, 30 years old).

### Peripheral blood mononuclear cell isolation, stimulation, and flow cytometry

Peripheral blood mononuclear cells (PBMCs) were isolated by a discontinuous density gradient (Nycomed, Zürich, Switzerland), counted by Trypan Blue (Sigma-Aldrich, St. Louis, MO, USA) exclusion, and resuspended in Roswell Park Memorial Institute (RPMI) 1640 (Thermo Fisher Scientific, Waltham, MA, USA) supplemented with 5% foetal bovine serum (FBS), 1% glutamine, and 1% penicillin/streptomycin (Euroclone, Pero, Italy). Cells were seeded in 96-well round-bottom plates at a concentration of 2 × 10^5^ cells/well. PBMCs were exposed for 2 h to 1 µM BTKi evobrutinib (CID 71479709, provided by Merck, Darmstadt, Germany (10)) and then stimulated for 18 h with lipopolysaccharide (LPS; Sigma-Aldrich), IL1β (Thermo Fisher Scientific), and Granulocyte-macrophage colony-stimulating factor (GM-CSF) (R&D Systems, Minneapolis, MN, USA). Technical duplicates were measured for each experimental condition. Thereafter, cells were labelled for flow cytometry with fluorescent antibodies against CD3, CD19, CD14, CD25, CD80, CD11a/LFA α chain, CD11b/Mac1, CD49d/VLA4, CD163, and 7AAD for cell viability (all from BioLegend, San Diego, CA, USA). Samples were acquired at BD FACSCanto II (BD Biosciences, San Jose, CA, USA) and analysed using the FlowJo software (Tree Star Inc., Ashland, OR, USA). Thresholds for positive staining were fixed on the corresponding isotype controls.

### Generation of mice with inducible inactivation of myeloid BTK and bone marrow chimera

All procedures involving animals were authorized by the Institutional Animal Care and Use Committee of the San Raffaele Scientific Institute and the Italian General Direction for Animal Health at the Ministry of Health (protocol number 821/2021-PR). Mice were housed in the institutional facility providing constant temperature (22°C ± 1°C), humidity (50%), and a 12-h light/dark cycle. They had *ad libitum* access to food and water. Transgenic mice carrying a floxable allele for BTK (C57BL/6N Tac-Btk<tm1c) were obtained from the EUCOMM MUTANT MOUSE program (CNRS, Orleans Cedex 2, France), while CX3CR1CreERT2iresGFP mice (B6.129P2(Cg)-CX3CR1tm2.1(cre/ERT2)Litt/WganJ) were purchased from Jackson Laboratory (MGI:J:190965) and backcrossed on the C57BL/6N mouse strain. Mice were genotyped by PCR using the following primers: BTKf/f mice: L3f:GCTGTGTGACTAAGCACCAA; L3r: CTGTAGACCAGGTAGGCCTCAA. CX3CR1CreERT2iresGFP mice: F: AAGACTCACGTGGACCTGCT; R: AGGATGTTGACTTCCGAGTTG; CRE: CGGTTATTCAACTTGCACCA. Breeding of the two strains generated the double-transgenic mice CX3CR1CreERT2iresGFP/BTKf/f (hereafter named BTK cKO) and BTKf/f controls.

To generate bone marrow chimera, C57BL/6L Y5.1 (CD45.1) recipient females were sub-lethally irradiated (9 Gy split into two doses) and then intravenously injected with 4 × 10^6^ total bone marrow CD45.2 cells collected from the tibia and femur of transgenic female mice carrying either CX3CR1CreERT2iresGFP/BTK f/f or BTK f/f alleles. Donor cells were isolated from 6–10-week-old mice, and recipient mice were 8 to 12 weeks old. Transplanted mice were kept under gentamycin (80 mg dissolved in drinking water) treatment for 2 weeks. Engraftment was evaluated 2 months after transplantation by flow cytometry on blood samples using antibodies against CD45.1 and CD45.2 antigens (BD).

Tamoxifen (TAM; Sigma) was dissolved in ethanol at a concentration of 200 mg/mL and then diluted 10 times in corn oil (Sigma). In the first experimental setting, mice were treated with 4 mg/die/mouse for 3 days. Next, the protocol was implemented with two series of 4 mg/die TAM for three consecutive days, 1 week apart. Bone marrow chimeric mice received 2 mg/die TAM for four consecutive days starting on experimental autoimmune encephalomyelitis (EAE) onset.

### Determination of BTK mRNA levels in myeloid cells

Spinal cords from TAM-treated control and BTK cKO mice were mechanically dissociated to obtain single-cell preparations, and CD11b-positive and CD11b-negative cells were purified by magnetic isolation (Miltenyi Biotec, Bergisch Gladbach, Germany). Total RNA was extracted by RNeasy Mini Kit (Qiagen, Hilden, Germany) according to the manufacturer’s recommendations, including DNase (Promega, Madison, WI, USA) digestion. cDNA synthesis was performed using the ThermoScript RT-PCR System (Invitrogen, Carlsbad, CA, USA) and Random Hexamer (Invitrogen), according to the manufacturer’s instructions. Real-time PCR was conducted using EvaGreen Supermix (Bio-Rad, Hercules, CA, USA) and the following primers: BTK f: AGCCTCTTCCCCCTACCCCAG; BTK r: GTTCATTGGCATGTAATCATAAAGGG; H3 f: GTGAAGAAACCTCATCGTTACAGGCCTGGTAC; H3 r: CTGCAAAGCACCAATAGCTGCACTCTGGAAGC.

### Western blotting

BTK cKO and control mice were perfused with saline and sacrificed with an overdose of ketamine/xylazine. Spinal cords and lymph nodes were dissected and homogenized in the following lysis buffer: 10 mM Tris-HCl pH 8, 1 mM EDTA pH 8, 100 mM NaCl, and 1% NP40 buffer supplemented with protease inhibitor cocktail (Sigma). Protein extracts were obtained using a tight-fitting glass Potter tissue grinder (1 mL; Wheaton) and then sonicated at a frequency of 20 kHz (10 times, 1 s). Forty micrograms of protein extract was loaded on 10% Mini-PROTEAN TGX Stain-Free precast gels for polyacrylamide gel electrophoresis (PAGE) (Bio-Rad). Upon electrophoresis, gels were transferred to nitrocellulose membranes (Millipore, Billerica, MA, USA) according to the manufacturer’s instructions. Blots were blocked in 5% Bovine serum albumin (BSA) in Tris-buffered saline plus 0.1% Tween-20 (TBS-T) for 1 h before receiving primary antibodies: αNf-H 1:1,000 (Thermo Fisher) and αBTK 1:200 (EPR20445, Abcam, Cambridge, UK) overnight at +4°C. Membranes were incubated with horseradish peroxidase (HRP)-labelled secondary antibodies (Bio-Rad) for 2 h at room temperature before being exposed to Clarity Max ECL (Bio-Rad). Images were acquired on ChemiDoc (Bio-Rad).

### Experimental autoimmune encephalomyelitis

EAE was induced in 8-week-old wild-type C57BL/6J (Harlan Laboratories, Indianapolis, IN, USA), transgenic, or bone marrow chimeric (generated in our lab as described above) female mice by subcutaneous injection of 200 µg MOG35–55 peptide emulsified in complete Freund’s adjuvant containing 5 mg/mL *Mycobacterium tuberculosis* (BD Biosciences). *Bordetella pertussis* toxin (Quadratech Diagnostics, Eastbourne, UK) was administered by intra-peritoneal (i.p.) or i.v. injection on the day of immunization (400 ng/mouse) and 2 days later (400 ng/mouse). Animals were monitored daily and scored as follows: 0 = no disease, 1 = flaccid tail, 2 = gait disturbance, 3 = complete hind limb paralysis, 4 = tetraparesis, and 5 = death as previously described (11). Eventually, 10 mg/kg/day of the BTKi evobrutinib (10) or vehicle was orally administered to immunized mice starting 3 days after disease onset. Experimenters were blind to the treatment regimen or genotype of EAE mice.

### Spleen and CNS cell preparation, flow cytometry analysis, and proliferation assay

Mice were anaesthetized, perfused with phosphate-buffered saline, and sacrificed. Spleens were mechanically dissociated, and red cells were lysed in ammonium chloride potassium lysis buffer (Sigma-Aldrich). Splenocytes were resuspended in RPMI 1640 (Thermo Fisher Scientific) supplemented with 10% FBS, 1% Pen-Strep, 1% sodium pyruvate, and 2% l-glutamine (Thermo Fisher Scientific) and shortly stimulated with 100 ng/mL LPS. Spinal cords, brain stem, and cerebellum were collected, and tissues were homogenized using a neural tissue dissociation kit according to the manufacturer’s procedures (Miltenyi Biotec). CNS cells were fractionated from myelin in 30% (volume/volume) isotonic Percoll and centrifuged for 20 min at 350 g without brake. Myelin ring was discarded, and CNS mononuclear cells were washed extensively with phosphate-buffered saline (PBS) and resuspended in RPMI with 10% FBS. To analyze TNF-α production, spleen or CNS myeloid cells were cultured for 4 h in the presence of GolgiPlug (BD Biosciences) and then stained with the following monoclonal antibodies specific for mouse surface antigens: CD45, CD11b, Ly6C, CD49d, and TNF-α (all from BioLegend) and CD206 and CD163 (all from Invitrogen). Cells were fixed, permeabilized using the Cytofix/Cytoperm Plus kit (BD Biosciences), and stained with anti-TNF-α (BD Biosciences). Cytofluorimetric measurements were performed using the FACSCanto II flow cytometer (BD Biosciences), and FlowJo (Tree Star Inc., Ashland, OR, USA) was used for data analysis.

For proliferation assays, splenocytes were seeded in 96-well round-bottom plates in complete RPMI medium and stimulated with increasing concentrations of MOG35–55 peptide. After 72 h, cultures were pulsed for 18 h with 0.5 mCi/well of [^3^H]thymidine and harvested. Thymidine incorporation was measured from quadruplicate cultures per condition on a β-counter (PerkinElmer, Waltham, MA, USA), and data were reported as proliferation index (cpm + antigen/cpm media).

### Neuropathology

Mice were perfused transcardially with 4% paraformaldehyde in PBS pH 7.2. Upon post-fixation, spinal cords were collected, processed, and sectioned as previously described (12). Briefly, spinal cord sections (20 µm thick) encompassing 2.5 mm of the lumbar tract were used for the neuropathological assessment of demyelination and axonal damage by Luxol fast blue (LFB) and neurofilament (NF) staining, respectively. For Nf labeling, cryosections were rinsed in 1× PBS, incubated in 3% H_2_O_2_ for 20 min, and blocked with a solution of 10% FBS, 1 mg/mL BSA, and 0.1% Triton X-100 in PBS for 1 h at room temperature. Anti-neurofilament heavy chain (Nf) antibodies (1:100, Thermo Fisher) were diluted in the blocking mix and incubated at 4°C overnight. The following day, sections were rinsed in 1× PBS before applying the secondary antibody (biotin-conjugated, Vector Laboratories Inc., Burlingame, CA, USA) for 2 h. Then, sections were washed in 1× PBS before adding the ABC kit (Vector Laboratories). Immunohistochemistry was revealed by incubating slices with DAB solution. Densities of Nf fibers were calculated in the ventral corticospinal tract.

### Statistics

Statistical analyses were performed in Excel or GraphPad Prism. Normality of data distribution was assessed by the Kolmogorov–Smirnov statistics or D’Agostino and Pearson omnibus normality test. Student’s t-test (in case of normal distribution) or non-parametric Mann–Whitney U-test (in case of non-normal distribution) was performed to compare means. For the statistical evaluation of EAE score and proliferation assays, linear regression analyses with 95% confidence interval and/or the Mann–Whitney U-test were used.

## Results

### BTKi may support expression of CD49d and CD163 in human monocytes under inflammatory conditions

**Figure 1:**
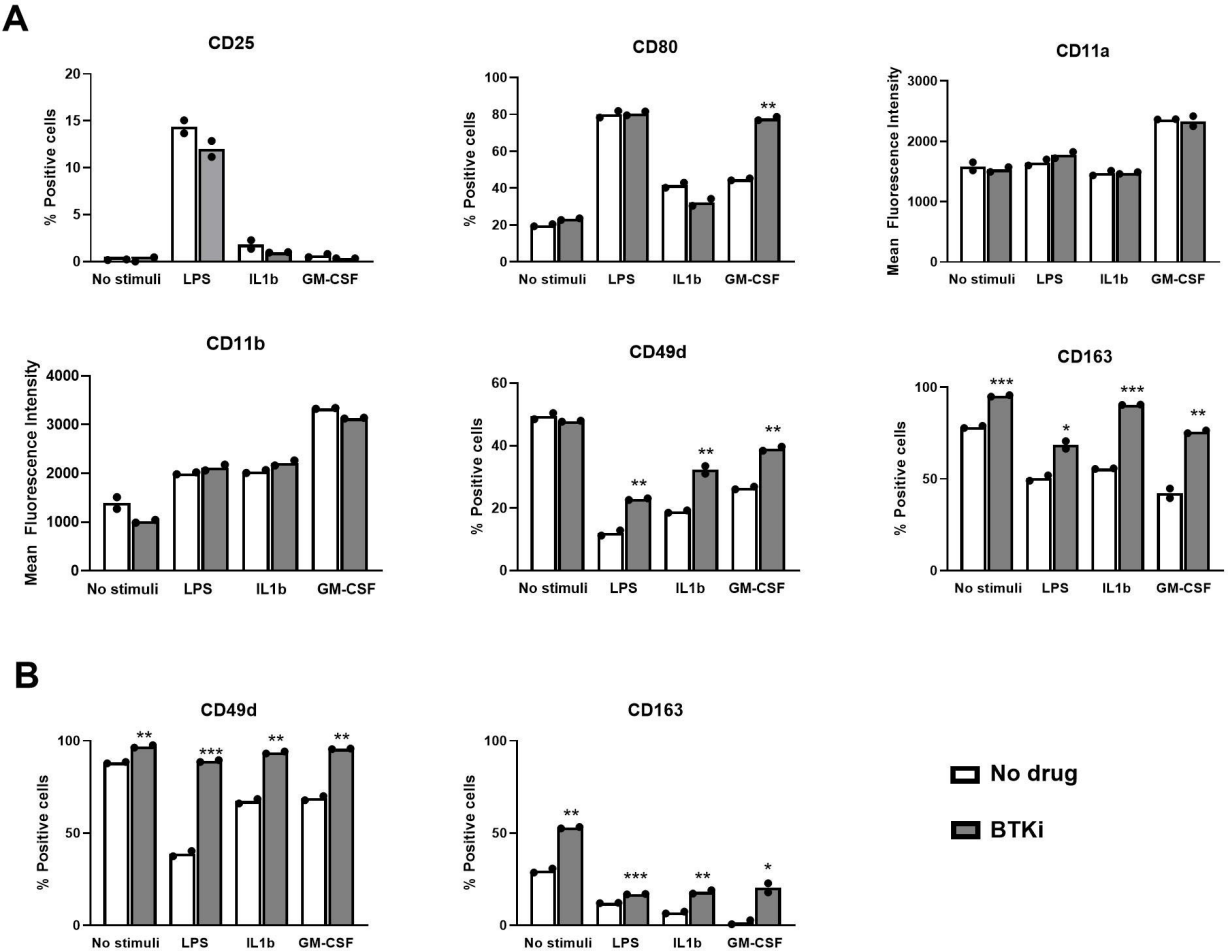
BTKi alters monocyte markers under distinct inflammatory conditions. Expression of monocyte markers in PBMC cultures from a single healthy **(A)** or MS **(B)** subject. PBMCs were exposed to vehicle or 1 µM BTKi under distinct inflammatory conditions. Representative data are shown from three performed experiments for each condition. Bars indicate mean, and dots indicate technical replicates. *p-value < 0.05; **p-value < 0.01; ***p-value < 0.001. BTKi, Bruton’s tyrosine kinase inhibitor; PBMC, peripheral blood mononuclear cell; MS, multiple sclerosis.

Assuming that daily treatment with BTKi may target circulating monocytes, to evaluate the impact of the pharmacological inhibition of BTK on these human myeloid cells under resting and inflammatory conditions, we exposed freshly isolated PBMCs from healthy subjects to 1 µM BTKi for 2 h and then to inflammatory stimuli (300 pg/mL LPS, 10 ng/mL IL1β, and 10 ng/mL GM-CSF) or vehicle for 18 h, stained for markers of distinct myeloid cell functions (CD25 for activation; CD80 for co-stimulation; CD11a/LFA α chain, CD11b/Mac1, and CD49d/VLA4 for adhesion; and CD163 for phagocytosis) or with isotype controls, and finally acquired cells by flow cytometry. As shown in a representative experiment relative to PBMC cultures established from one healthy subject (**Figure 1A**), BTKi did not impact the induction of CD25 resulting from inflammatory stimulation at the tested dose, while it enhanced the proportion of CD80-positive monocytes under GM-CSF stimulation (**Figure 1A**). Among adhesion markers, basal and induced CD11a and CD11b levels were not affected by BTKi (**Figure 1A**), while CD49d, which was downregulated by inflammatory mediators, was partly maintained if monocytes were exposed to BTKi (**Figure 1A**, **Supplementary Figure S1A**). Similarly, BTKi significantly counteracted CD163 downregulation induced by all inflammatory mediators under investigation (**Figure 1A**, **Supplementary Figure S1A**). We then repeated the treatment using PBMCs from untreated MS subjects to assess the levels of CD49d and CD163 on monocytes in both unstimulated and stimulated conditions. As shown in a representative experiment in **Figure 1B** and reproduced in additional experiments (**Supplementary Figure S1B**), BTKi enhanced the expression of CD49d and CD163 levels in both experimental settings. We can conclude that BTKi sustains CD49d and CD163 expression in healthy and MS monocytes under inflammatory conditions.

### BTKi ameliorates EAE expression and reduces CNS-infiltrating inflammatory macrophages

**Figure 2:**
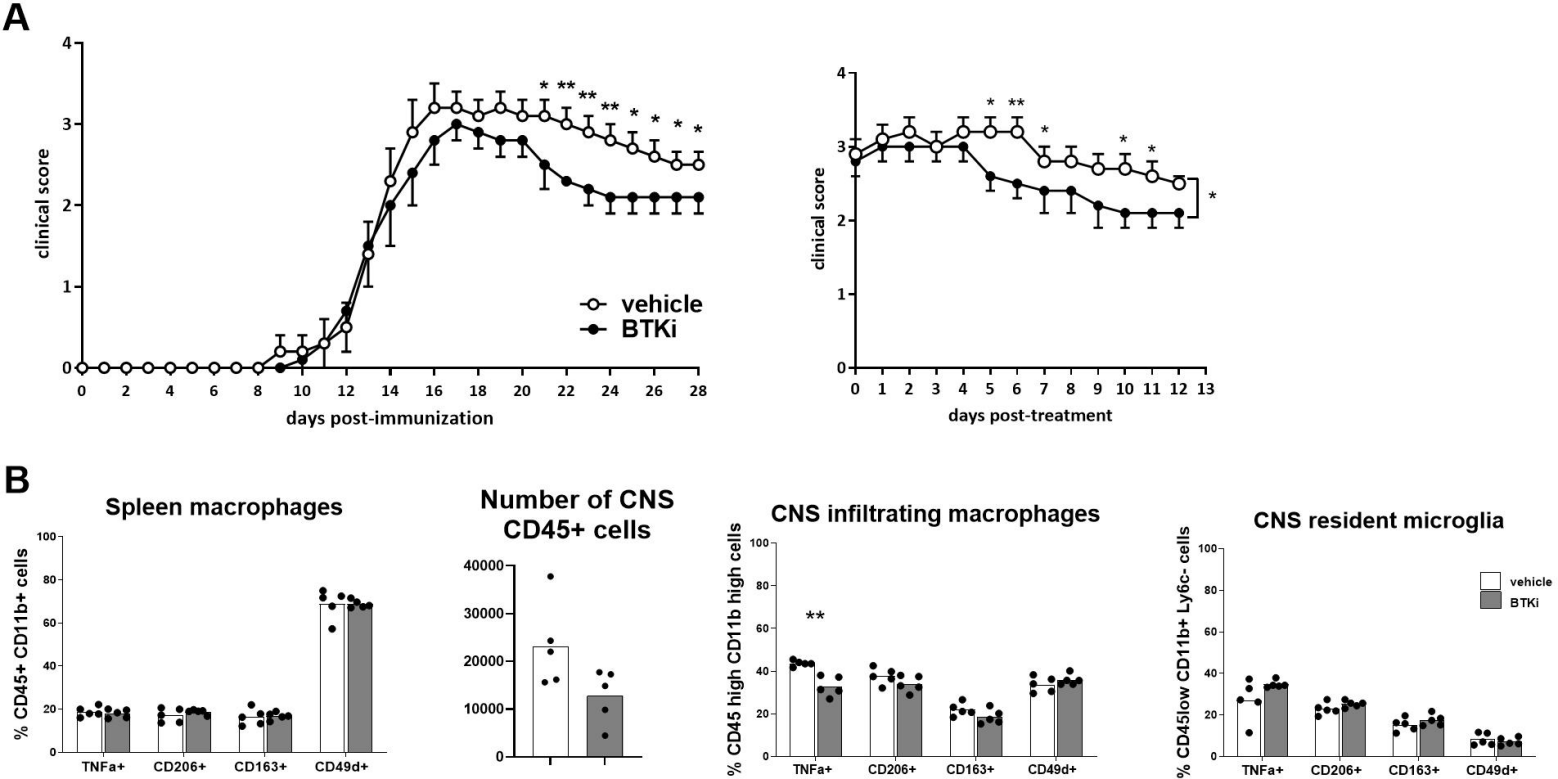
BTKi alters EAE expression and myeloid cell phenotype. **(A)** EAE expression in vehicle-treated and BTKi-treated mice (n = 11–13) according to days post-immunization and days post-treatment. Oral treatment with 10 mg/kg/day BTKi or vehicle was administered starting the third day after disease onset. Data are shown as mean ± standard error of the mean (SEM). **(B)** Myeloid cell phenotype in spleen, number of CD45-positive cells, and myeloid cell phenotype in CNS of vehicle- or BTKi-treated mice. Bars indicate mean, and dots indicate distinct mice. *p-value < 0.05; **p-value < 0.01. BTKi, Bruton’s tyrosine kinase inhibitor; EAE, experimental autoimmune encephalomyelitis; CNS, central nervous system.

EAE was induced in wild-type C57BL/6J mice, and oral treatment with 10 mg/kg/day BTKi or vehicle was administered starting the third day after disease onset. Despite no weight difference being observed between BTKi- and vehicle-treated mice, significant clinical score amelioration emerged in EAE mice treated with BTKi (**Figure 2A**). Representative EAE animals of each group were sacrificed at day 28 post-immunization (p.i.); the spleen and CNS were extracted, dissociated, and labelled for flow cytometry assessment of the inflammatory marker TNF-α, the anti-inflammatory marker CD206, phagocytic receptor CD163, and the adhesion molecule CD49d on myeloid cells. As shown in **Figure 2B**, no difference in the expression of these markers was detected in spleen macrophages between the two groups of animals. When looking at CNS-resident microglia (CD45low CD11b+ Ly6C−) and CNS-infiltrating macrophages (CD45high CD11b+), no difference was detected between the two groups of animals except for the significant reduction of TNF-α-producing inflammatory macrophages in BTKi-treated animals (**Figure 2B**).

### BTK in myeloid cells does not support experimental neuroinflammation

**Figure 3:**
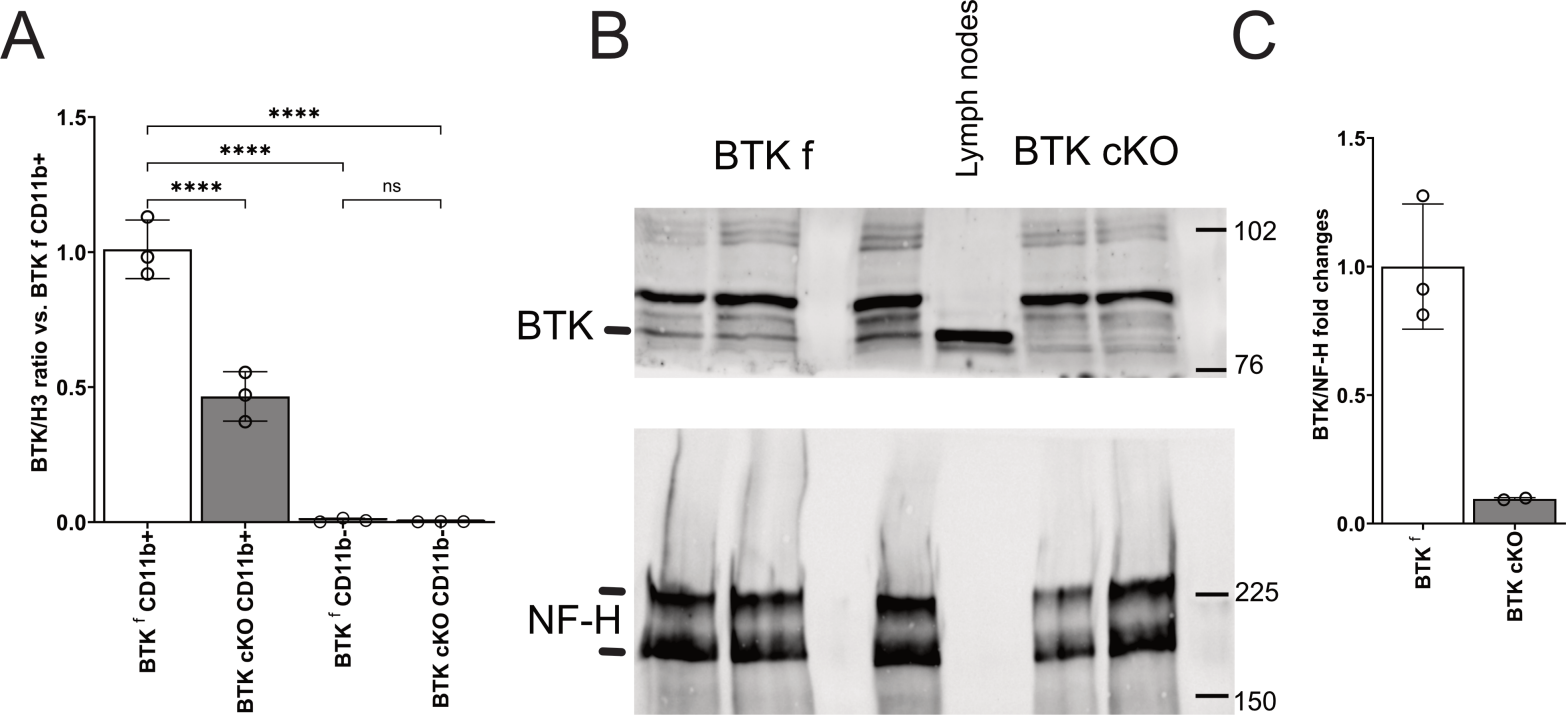
**(A)** Quantitative PCR for BTK in CD11b+ and CD11b− cells sorted from the brains of control BTKf and BTK cKO mice. Dots indicate individual mice; columns show mean ± SD. **(B, C)** Western blotting for BTK (**B**, upper panel) and NF-H (**B**, bottom panel) in the CNS of control BTKf and BTK mice and relative quantification **(C)**. Lymph node protein extract of a BTKf animal was included in B for BTK protein reference. ****p < 0.0001; ns, not significant. BTK, Bruton’s tyrosine kinase; CNS, central nervous system.

To understand the contribution of BTK in myeloid cells to neuroinflammation, mice with inducible inactivation of myeloid BTK (BTK cKO mice) were generated by crossing transgenic animals carrying floxable Btk alleles with CX3CR1CreERT2iresGFP mice carrying the inducible CRE under the CX3CR1 promoter. Initially, double-transgenic mice and controls were injected with TAM (4 mg/die/mouse) for three consecutive days starting from P60 and sacrificed at P90. Quantitative PCR experiments measuring BTK mRNA levels in purified CD11b-positive or CD11b-negative brain cells did not detect any BTK signal in CD11b− cells while evidencing robust BTK transcript expression in CD11b+ cells of BTKfl control mice (**Figure 3A**). BTK mRNA was halved in CD11b+ cells from BTK cKO mice (**Figure 3A**). To further reduce BTK levels in BTK cKO mice, the TAM protocol was implemented by adding three more TAM injections after a 1-week washout from the first series. Mice were sacrificed 3 weeks after the last TAM injection, and spinal cords were collected to assess BTK expression. Having established that CD11b− cells do not express BTK (**Figure 3A**), BTK levels were quantified in protein extracts from the whole spinal cord and included lymph node extracts from a BTKfl control as a reference for BTK expression and staining for neurofilament heavy chain as quality control for CNS protein extracts. As shown in **Figure 3B**, the BTK band detected in the lymph node was present in spinal cord extracts from control mice and was 10-fold lower in those from TAM-treated BTK cKO mice. These results indicate that CreERT2-mediated recombination of the BTK locus was efficient in myeloid cells and that the main source of BTK within the CNS was microglia.

**Figure 4:**
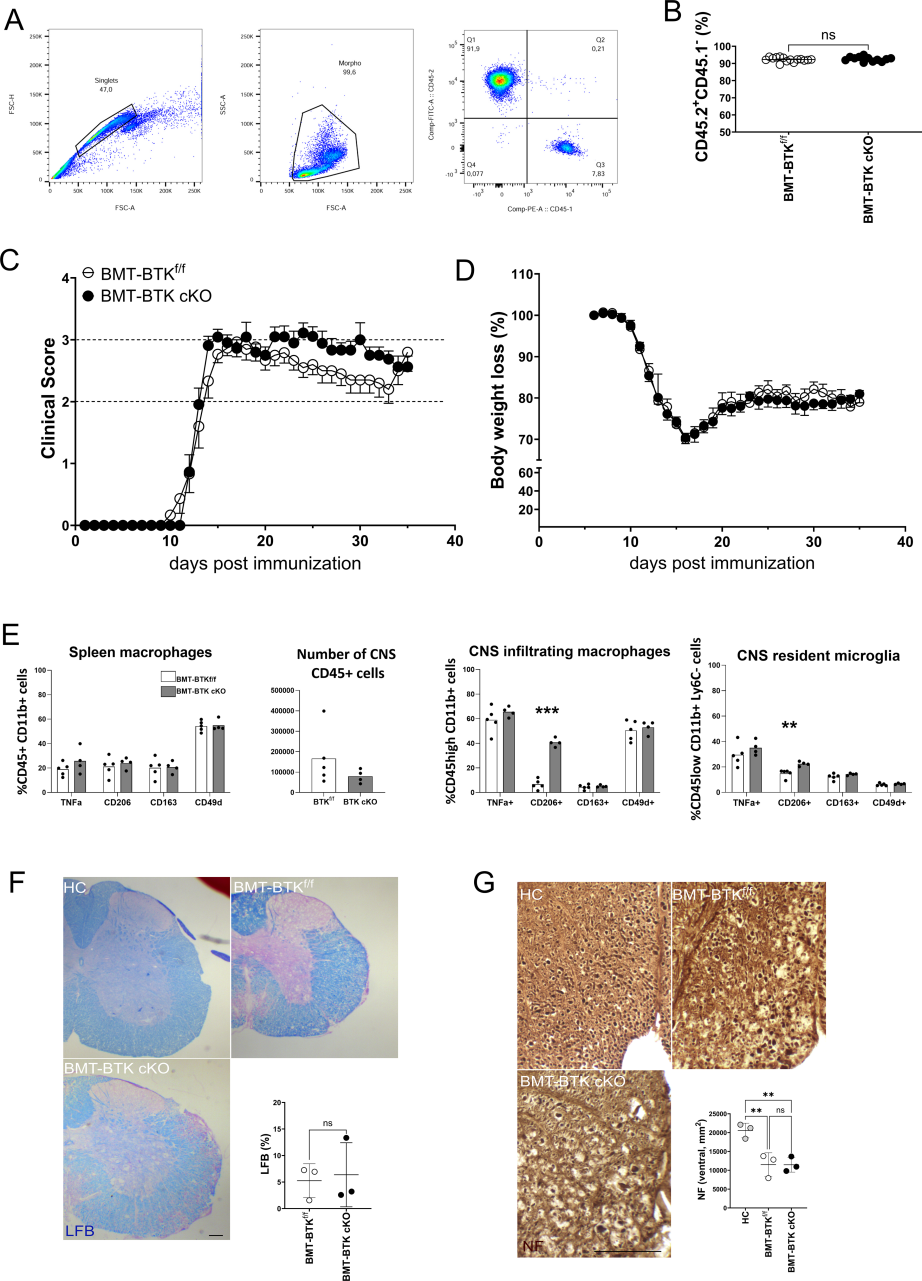
BMT-BTK cKO mice do not display alterations in EAE course and neuropathology despite the higher frequency of CD206-positive myeloid cells in the CNS. **(A)** Representative flow cytometry plots for CD45.1 and CD45.2 detection in mouse blood samples. **(B)** Levels of cell engraftment in transplanted animals (each dot represents a single animal). **(C, D)** Clinical course of EAE **(C)** and body weight changes **(D)** in BMT-BTKf/f (n = 15) and BMT-BTK cKO mice (n = 11). Variations in panel D are calculated as percentages of body weight measured on the day of immunization. Data are shown as mean ± SEM. **(E)** Myeloid cell phenotype in spleen, number of CD45-positive cells, and myeloid cell phenotype in CNS of BMT-BTKf/f or BMT-BTK cKO mice (n = 4–5) are also reported. Bars indicate mean, and dots indicate distinct mice. **(F)** Myelin content in the lumbar spinal segment of BMT-BTKf/f and BMT-BTK cKO mice by LFB staining. Control sections from healthy control (HC) mice were included in each experiment. Quantification of demyelinated areas (% of the section) is shown in the graph. **(G)** Axonal damage in the lumbar spinal segment of BMT-BTKf/f and BMT BTK cKO mice by NF staining. NF+ axons were scored in the corticospinal tract and tectospinal tract. Dots in panels F and G indicate individual animals, while lines indicate mean and SD. **p-value < 0.01; ***p-value < 0.001; ns, not significant. Scale bars, 100 µm. BMT, bone marrow transplanted; BTK, Bruton’s tyrosine kinase; EAE, experimental autoimmune encephalomyelitis; CNS, central nervous system; LFB, Luxol fast blue.

EAE immunopathogenesis is largely caused by infiltrating myelin-reactive T cells and macrophages, with the latter also expressing CX3CR1 (13). To assess the role of BTK in short-lived circulating CX3CR1-positive cells, bone marrow transplanted (BMT) chimeric mice were generated by reconstitution of irradiated CD45.1+ C57BL/6J mice with CD45.2+ bone marrow cells derived from female BTK cKO or control littermates, and engraftment was verified 2 months later by flow cytometry for CD45.1 and CD45.2 antigens in blood cells. Percentages of engraftment were above 90% in all mice independently of the genotype of transplanted bone marrow cells (**Figures 4A, B**). Then, EAE was induced in BMT chimeric mice, and 2 mg/die TAM was administered for four consecutive days starting at disease onset. As shown in **Figure 4C**, BMT-BTK cKO displayed similar clinical scores to those of controls over time, with an apparently more severe EAE course after 20 days post-immunization (dpi), which was not significant. Body weight loss did not allow for discrimination between BMT-BTK cKO mice and the control group (**Figure 4D**). These results suggest that BTK expressed by short-lived CX3CR1-positive immune cells does not support EAE expression. At the end of the EAE experiment, spleen and CNS cells were isolated from representative animals of each group, labelled, and analysed by flow cytometry. Phenotypic characterization of inflammatory (TNF-α-positive), anti-inflammatory (CD206-positive or CD163-positive), and migratory (CD49d-positive) myeloid cells did not indicate any change in the spleen and the CNS except for a higher frequency of CD206-positive infiltrating macrophages and resident microglia in BMT-BTK cKO mice (**Figure 4E**). Finally, spinal demyelination and axonal damage were assessed by labeling sections with LFB and NF, respectively. Demyelination levels in BMT-BTK cKO EAE mice did not differ from those in controls (**Figure 4F**). The densities of NF+ fibers scored in the ventral spinal cord were significantly reduced in EAE mice compared to naïve mice, but no difference emerged between BMT-BTK cKO and control EAE mice (**Figure 4G**).

**Figure 5:**
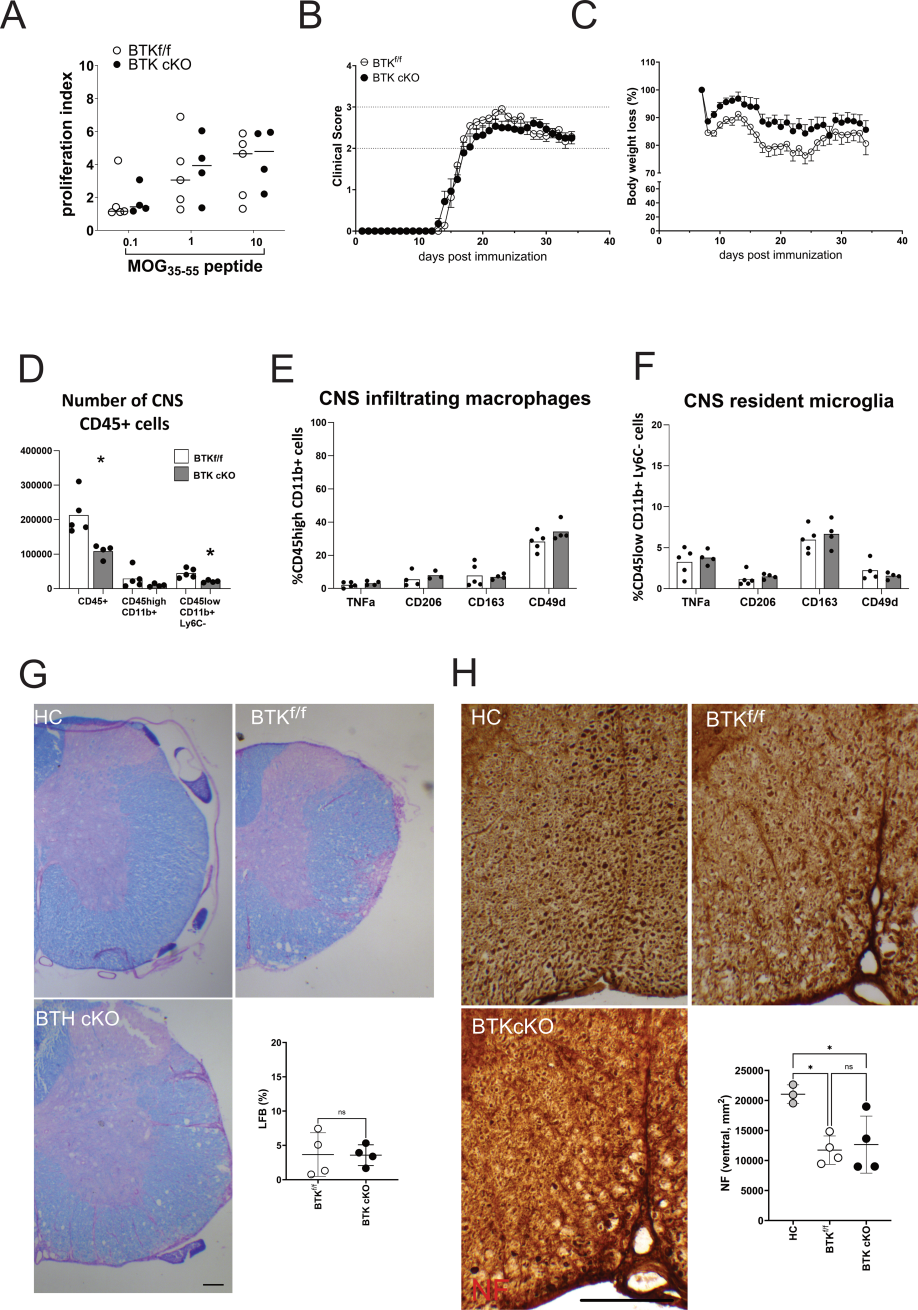
BTK inactivation in long-lived CX3CR1+ cells does not alter EAE progression. **(A)** Myelin-reactive T-cell proliferation measured *ex vivo* in splenocytes from BTKf/f or BTK cKO mice. Each dot represents an individual mouse. **(B, C)** Clinical course of EAE **(B)** and body weight loss **(C)** in BTKf/f (n = 11) and BTK cKO mice (n = 14). Data are shown as mean ± SEM. **(D–F)** Number of CNS-infiltrating CD45-positive cells **(D)** and phenotype of macrophages **(E)** or microglia **(F)** (n = 4–5). Bars indicate mean, and dots indicate distinct mice. **(G)** Myelin content in the lumbar spinal segment of BTKf/f and BTK cKO mice by LFB staining. Control sections from healthy control mice were included in each experiment. Quantification of demyelinated areas (% of the section) is shown in the graph. **(H)** Axonal damage in the lumbar spinal segment of BTKf/f and BTK cKO mice by NF staining. NF+ axons were scored in the corticospinal tract and tectospinal tract. Dots indicate individual animals, while lines indicate mean ± SD. *p < 0.05; ns, not significant. Scale bars, 100 µm. BTK, Bruton’s tyrosine kinase; EAE, experimental autoimmune encephalomyelitis; CNS, central nervous system; LFB, Luxol fast blue.

Next, an established TAM protocol that allows for gene inactivation in microglial cells was applied by leveraging the different turnover rates between microglia and circulating CX3CR1+ immune cells (14, 15). After TAM treatment (4 mg/mouse for 3 days, 1-week washout, 4 mg/mouse for further 3 days), BTK cKO and control mice were maintained for 2 months before EAE immunization to allow for the replacement of fast renewing circulating immune cells with new cells deriving from bone marrow precursors while maintaining gene ablation in long-lasting and slowly dividing microglia. After EAE induction, the generation of encephalitogenic T cells in lymphoid organs, disease expression, myeloid cell phenotype, and demyelination were monitored. Some immunized animals were sacrificed at 9 dpi to measure the splenic output of myelin-reactive T cells before disease onset. As expected, no difference between BTK cKO and control mice emerged (**Figure 5A**). Both groups of animals displayed disease onset 14–16 days after immunization and developed similar chronic EAE curves with a slight, but not significant, reduction of body weight loss in BTK cKO mice (**Figures 5B, C**). The number of CD45-positive cells in the CNS of BTK cKO mice was significantly lower than that of control mice and was partly ascribed to the microglial population (**Figure 5D**). Phenotypes of CNS myeloid cells (as measured by flow cytometry) and neuropathological outcomes (as measured by LFB and NF staining) were not different between BTK cKO and control mice (**Figures 5E–H**).

## Discussion

This study sought to investigate the impact of BTK inhibitor evobrutinib on myeloid cell phenotype and the contribution of BTK in myeloid cells to experimental neuroinflammation.

The first observation regards the action of BTK inhibitors on markers of cell activation, co-stimulation, adhesion, and phagocytosis detected on human monocytes exposed *in vitro* to an inflammatory challenge. Monocytes from XLA patients do not display gross alterations in monocyte markers, such as CD11b, and functions like oxidative burst and phagocytosis induced *in vitro* by opsonized bacteria (16), suggesting that BTK may be dispensable or bypassed for such cell functions. Further, contrasting results have been published about the role of BTK in supporting inflammatory cytokine release by human myeloid cells from XLA subjects following Toll-like receptor stimulation (17, 18). Regarding the impact of BTKi on specific markers in healthy immune cells, CD25 upregulation, which represents a proxy for innate and adaptive immune cell activation, can be inhibited by BTK inhibitors in B cells (19) but not monocytes (as shown in this study). The same applies to the levels of the costimulatory molecule CD80, which can be limited by BTKi in activated B cells (20) but not monocytes (this study). Further, we do not detect any effects of BTKi on CD11a and CD11b levels in human monocytes after LPS or cytokine exposure. Regarding effects of BTK inhibition on the expression of CD49d or CD163, no information is available for healthy human immune cells, while it is known that CD49d is expressed in approximately 40% of chronic lymphocytic leukaemia, a B-cell malignancy, and contributes to resistance to BTKi treatment (20, 21) and that CD163 expression in tumour-infiltrating macrophages or circulating soluble CD163 is associated with poor prognosis in mantle cell lymphoma (22). Here, we showed that under inflammatory conditions, BTKi may strongly support the maintenance of integrin CD49d, which is essential to direct immune cell trafficking towards the CNS (23), and of phagocytic receptor CD163, which may promote an anti-inflammatory program within the CNS via ingestion of myelin debris (24). This qualitative *in vitro* evidence is also reproduced with cells from a small number of untreated MS patients and suggests candidate immunological markers for quantitative *ex vivo* drug monitoring in large prospective studies.

Interestingly, BTKi may support *in vitro* differentiation of human monocytes into macrophages expressing anti-inflammatory markers such as CD163 and CD206 (25), raising the possibility that treatment with BTKi may promote the generation of CNS-infiltrating myeloid cells exerting protective functions. Further, BTKi can be detected in the cerebrospinal fluid of treated MS patients (26), indicating that it can potentially act on CNS-resident cells, including microglia. Indeed, BTK inhibition dampens microgliosis in LPS-induced neuroinflammation and chronic white matter ischemia (27, 28) and enhances microglial phagocytosis while reducing microglial TNF-α *in vivo* in the animal model of neuromyelitis optica (29). Therapeutic administration of BTK inhibitors has beneficial effects on the clinical expression of EAE in SJL/J (30), Biozzi (31), and C57BL/6 mice [ (32) and our study], while ineffective when started in the chronic phase of disease in C57BL/6 mice (10). The assessment of myeloid cell phenotype is not available for the first model, while it indicates modest reductions in IL1β and CD206 levels in infiltrating myeloid cells and microglia in BTKi-treated Biozzi mice (31). Single-cell RNA-seq analysis relative to spinal cords from BTKi- or vehicle-treated EAE C57BL/6 mice indicates reversal of EAE-associated markers such as Csf-1 and Trem2 (32). Our flow cytometry experiments detected a mild decrease in proinflammatory CNS-infiltrating macrophages but not microglia and no difference in the frequency of anti-inflammatory myeloid cells in BTKi-treated and control EAE C57BL/6 mice. Reductions in proinflammatory markers on myeloid cells persist also in the absence of clinical efficacy, when treatment is provided during chronic EAE (10), questioning the real contribution of myeloid cell phenotype changes under BTKi to disease control.

While the pharmacological inhibition of BTK *in vivo* may highlight associations between BTK signaling and cell phenotypes under disease, the definition of the functional link between BTK signaling and disease expression requires the adoption of genetic approaches in animal models. Conventional BTK-deficient mice display mild EAE expression compared to wild-type mice; however, the developmental defects in B-cell and myeloid compartments do not allow proper assessment of BTK contribution to mature immune function in a specific cell type during neuroinflammation (33, 34). To address the contribution of BTK signaling in myeloid cells to experimental neuroinflammation, we crossed mice carrying floxable BTK alleles with mice expressing the TAM-dependent Cre recombinase gene under the promoter of the G-protein coupled receptor CX3CR1. The expression atlas of CX3CR1 is quite complex, as it includes all circulating monocytes and also subsets of other cell types such as T cells and NK cells (35, 36). The expression of CX3CR1 in the CNS encompasses border-associated macrophages and virtually all homeostatic microglia (35, 37). Some literature reports about BTK protein expression in CNS cells as detected by immunofluorescence or flow cytometry in the absence of appropriate experimental controls (e.g (10).). Our experiments indicate BTK mRNA in CNS-isolated CD11b+ and not CD11b− cells and its efficient deletion in the CNS of the inducible double-transgenic mice. The analysis of bone marrow transplanted mice shows that BTK inactivation in circulating CX3CR1+ cells does not alter EAE course and neuropathology despite some increase in the frequency of anti-inflammatory CD206-positive myeloid cells in the CNS. Similarly, the adoption of a TAM protocol leveraging on the differences in myeloid cell turnover (14, 15) shows that BTK inactivation in long-lived myeloid cells, including microglia, has no impact on the generation of myelin-reactive T cells, EAE expression, phenotype of CNS-infiltrating or CNS-resident myeloid cells, and tissue damage.

Overall, our data demonstrate that although BTKi may induce phenotypic changes in myeloid cells and ameliorate experimental neuroinflammation, the specific contribution of BTK signaling in myeloid cells to the development and severity of EAE is negligible.

## Data Availability

The original contributions presented in the study are included in the article/**Supplementary Material**. Further inquiries can be directed to the corresponding authors.

## Supplementary material

Supplementary Figure 1 BTKi supports CD49d and CD163 expression in monocytes under distinct inflammatory conditions. For each experimental condition the ratio between the mean expression of monocyte markers in PBMC cultures with BTKi and that in parallel cultures without the drug was calculated for three healthy **(A)** or MS **(B)** subjects.
