# Supplementary figures and images for: The contribution of BTK signaling in myeloid cells to neuroinflammation
Source: Front Immunol. 2025 Jun 18;16:1595069. doi: 10.3389/fimmu.2025.1595069 (PMC12213430; doi:10.3389/fimmu.2025.1595069)

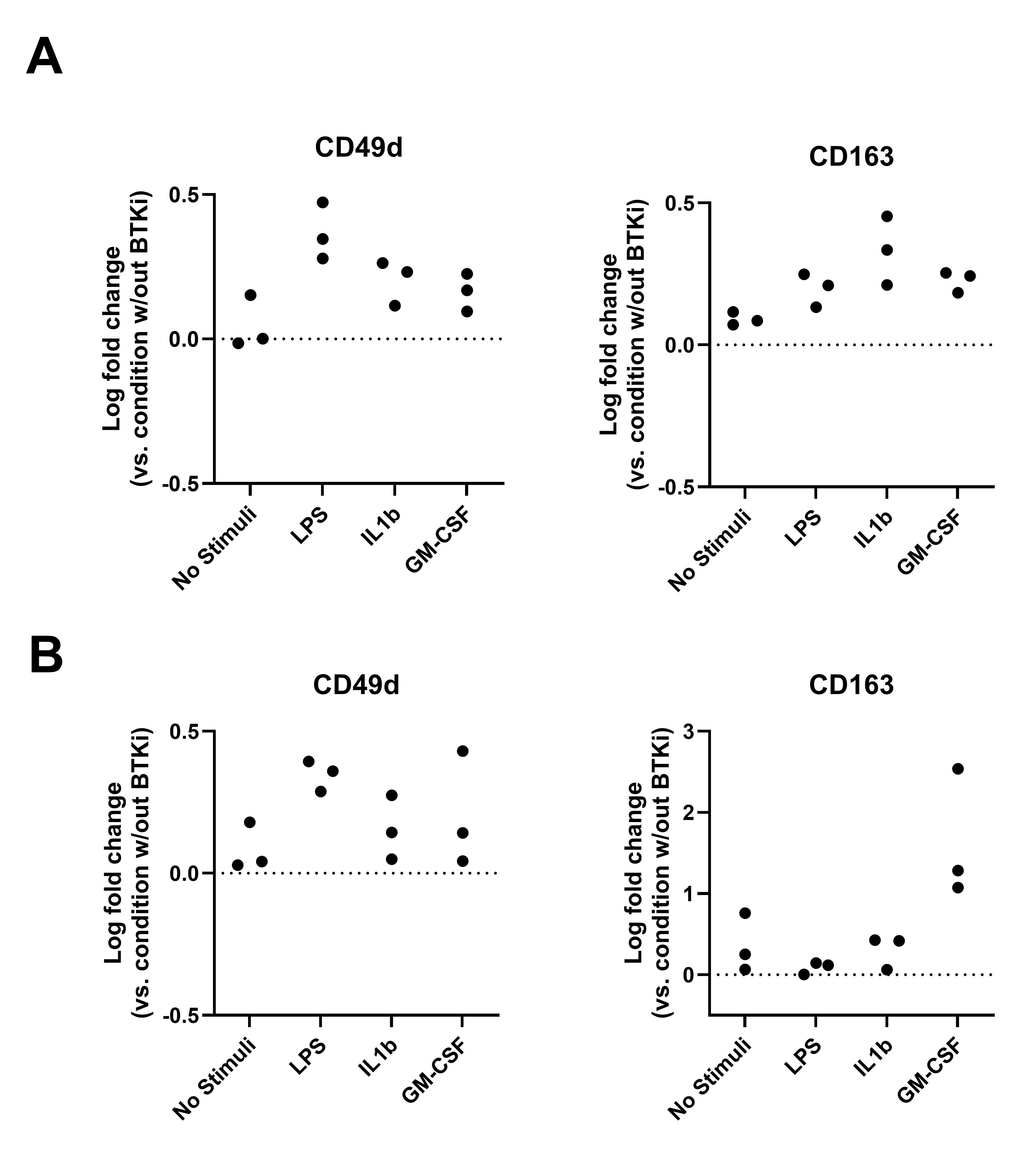

Supplement: Supplementary Figure 1 — BTKi supports CD49d and CD163 expression in monocytes under distinct inflammatory conditions. For each experimental condition the ratio between the mean expression of monocyte markers in PBMC cultures with BTKi and that in parallel cultures without the drug was calculated for three healthy (A) or MS (B) subjects. [file Image1.jpeg]
